# Microbial biofilm community structure and composition on the lithic substrates of Herculaneum Suburban Baths

**DOI:** 10.1371/journal.pone.0232512

**Published:** 2020-05-04

**Authors:** Antonino De Natale, Bruno Hay Mele, Paola Cennamo, Angelo Del Mondo, Mariagioia Petraretti, Antonino Pollio

**Affiliations:** 1 Dipartimento di Biologia, University of Naples Federico II, Complesso Universitario di Monte Sant'Angelo, Naples, Italy; 2 Department of Integrative Marine Ecology, Stazione Zoologica Anton Dohrn, Napoli, Italy; 3 Facoltà di Lettere, University Suor Orsola Benincasa of Naples, Naples, Italy; 4 Metodologie Analitiche per la Salvaguardia dei Beni Culturali (Masbc), Task Force d’Ateneo Federico II di Napoli, Complesso Universitario di Monte Sant'Angelo, Naples, Italy; International Center for Theoretical Physics - South American Institute for Fundamental Research, BRAZIL

## Abstract

In this work, we want to investigate the impact of different substrates and different environmental condition on the biofilm communities growing on plaster, marble, and mortar substrates inside the Herculaneum Suburban Baths. To do so, we measured environmental conditions and sampled biofilm communities along the walls of the baths and used culture-dependent and -independent molecular techniques (DGGE) to identify the species at each sampling sites. We used the species pool to infer structure and richness of communities within each site in each substrate, and confocal light scanning microscopy to assess the three-dimensional structure of the sampled biofilms. To gather further insights, we built a meta-community network and used its local realizations to analyze co-occurrence patterns of species. We found that light is a limiting factor in the baths environment, that moving along sites equals moving along an irradiation gradient, and that such gradient shapes the community structure, de facto separating a dark community, rich in Bacteria, Fungi and cyanobacteria, from two dim communities, rich in Chlorophyta. Almost all sites are dominated by photoautotrophs, with Fungi and Bacteria relegated to the role of rare species., and structural properties of biofilms are not consistent within the same substrate. We conclude that the Herculaneum suburban baths are an environment-shaped community, where one dark community (plaster) and one dim community (mortar) provides species to a “midway” community (marble).

## Introduction

The Roman city of Herculaneum was destroyed in AD 79 when the Vesuvius erupted and inundated the town with hot volcanic material, submerging houses and streets [1]. Due to their burial under a thick layer of solidified lava, the Suburban Baths of ancient Herculaneum are one of the best-conserved bathing complexes that survive from antiquity. The building that hosted the baths was probably built in the AD 40s [2] and laid at an intermediate level between the city and the former seashore line. The inside environment is highly humid, indirectly exposed to a weak light filtering from the outside, and thermally stable [3]; moreover, the public is currently not allowed to enter the site. These conditions allowed different microorganisms to quickly and permanently colonize the walls of the Baths, forming dark green or black-pigmented patinas and incrustations that extensively spread on different substrates in some of the Baths’ rooms.

Biological colonization is a complex dynamic, depending on both substrates and environmental factors [4,5]. Usually, the influence of the latter is stronger, but when light, relative humidity and temperature do not represent limiting factors, physicochemical characteristics of substrates become crucial drivers of the assembly of microbial communities [6]. Moreover, species composition may also vary in biofilm growing very close to each other, and, apparently, in the same chemical and physical conditions [7], suggesting the involvement of species interactions and stochasticity in the community assembly process. Thus, colonization can strongly depend on the organism that establishes the first firm relationship with the substrate, conditioning the subsequent steps of the biofilm consolidation [8]. Community-level interactions are unique to each structured community of microorganisms [9], and strongly intertwined with the architecture of the biofilm. At a fundamental level, spatial interactions among microorganisms forming biofilms on monuments can often explain important attributes of biofilms [10] and are well documented [11]. Actinobacteria and filamentous Cyanobacteria, for example, often grow in a close association, sometimes establishing a direct cell-to-cell contact and some other times sharing a matrix of extracellular polymeric substances (EPS). Such matrix represents at the same time the adhesion agent used by organisms to remain anchored to the substrate and the common ground that connects them. It also has a potential role in modulating chemical signals at the base of microorganisms' interactions [12].

This study aims to assess the influence of substrate and microclimatic conditions on species composition and three-dimensional structure of biofilms growing in Herculaneum Suburban Baths. To do so, we measured environmental variables and collected biofilms samples from three of the substrates used in building the Baths: plaster from the *Vestibulum* (on which are still present frescoes traces), marble from the *Tepidarium*, and mortar from the swimming pool. After collection, we identified the species present in each environmental sample by coupling culture-dependent and -independent techniques and used this information to assemble a co-occurrence matrix and proceeded to analyze the structure and the richness of the communities at various sites. Then we used confocal laser Microscopy (CLSM), the election tool for non-destructive analyses of biofilm on monuments [13,14], to elucidate the spatial organization of the communities. Finally, we transformed the co-occurrence matrix into a meta-community network analyzing both its local realizations (*i*.*e*. the sampled communities) and the meta-communities associated with the single substrates.

## Material and methods

### Environment description and sampling

**Fig 1:**
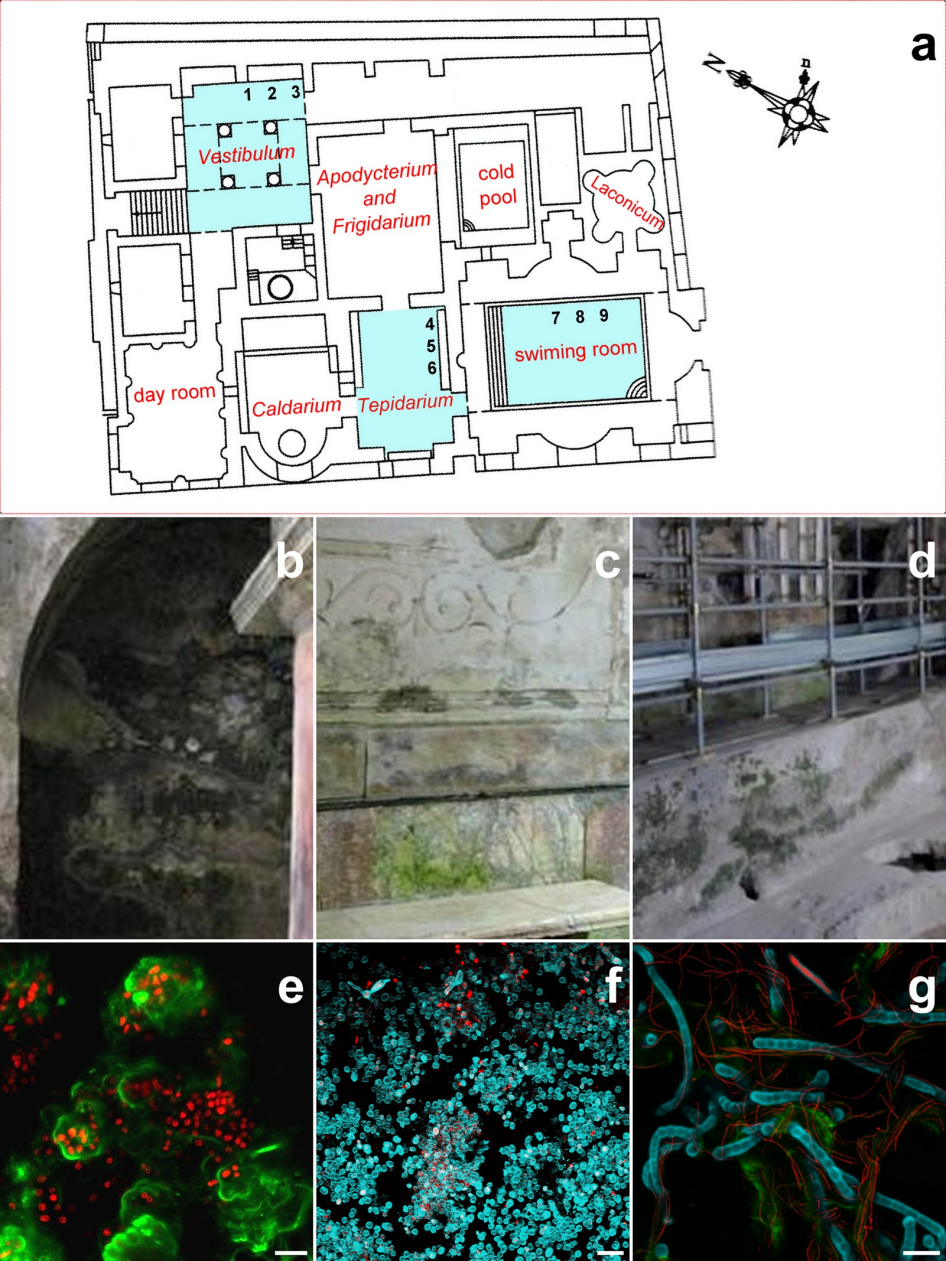
(a) Map of the Suburban Baths, with details of the *Vestibulum* (b), the *Tepidarium* (c), and of the room with a large heated swimming pool (d). Light blue highlights sampled rooms, with numbers marking the biofilm sampling sites. In following panels (e, f, g) are matched CLS-M microphotographs of sampled biofilms Scale bar 20 μm. Floor plan of suburban baths is not subject to copyright and has been de novo realized with freeware software Fiji.

The Herculaneum Baths architectural structure is the one generally used by Romans when building public baths (Fig 1A). The first chamber from the entrance is a *Vestibulum*, an ample room with four columns, delimitating a central square. The *Vestibulum* is followed by an *Apodyterium*, a *Frigidarium* and a room with a pool of cold water. Parallels to these rooms there is a day room open to the seaside landscape for conversation and relax, connected to the *Vestibulum*. Next to the “day room” is the *Caldarium*, then a room with decorations on the walls (*Tepidarium*) and finally a room with a large heated swimming pool. A *Laconicum*, used for saunas, is accessible from the heated pool room.

Among the Suburban Baths' rooms, only the *Vestibulum*, the *Tepidarium*, and the room with the large swimming pool were colonized by conspicuous biofilms, visible on three substrates: plaster in the *Vestibulum*, polychrome marble in the *Tepidarium*, and mortar in the swimming pool. Both plaster and mortar are non-homogeneous materials widely diffused in Herculaneum buildings: the former is made by a mixture of calcitic binder and volcanic scoriae, covered by a thin superficial layer of lime and marble powder [15], whereas the latter is prevalently composed by lime and inert volcanic aggregates with variable dimensions [16]. The general distribution of biofilms on the walls of the Suburban Thermae appeared patchy to the naked eye. Since the area is restricted to public, biofilm development and distribution is not affected by the presence of vectors. Sampling has been authorized by *Soprintendenza speciale per i beni archeologici Pompei*, *Ercolano e Stabia* (Via Villa dei Misteri, 1, 80045 Pompei, Naples, Italy) in the person of Arch. Giuseppe Zolfo, within an agreement with the laboratory of algal biology and ACUF collection at the Department of Biology, University of Naples “Federico II”.

In every site, we measured temperature and relative humidity using a thermo-hygrometer (model HI 9564, Hanna® Instruments, USA), and light intensity using a Climalux N light meter (Laboratori di Strumentazione Industriale s.p.a., Italy). pH on substrates was measured at sampling points using pH test paper strips, showing no consistent variation able to affect an homogeneous response of communities.

### Biofilm sampling

Biofilm samples were collected in situ during autumn 2016, using either sterile double-sided adhesive tape (1 cm wide) or by gently scraping the walls of the sampling sites with a sterile scalpel and depositing materials into sterile vials [17]; sampling was random and repeated three times per room/material (numbers in Fig 1A).

### Culture-dependent isolation of microbial strains

Each sample collected in the field with a sterile scalpel was suspended in a sterile isotonic solution (10 mg/ml). From this stock, serial dilutions up to 10^−7^ were prepared, and 10 μl from 10^−4^ or 10^−6^ dilutions were spread on 9 cm diameter Petri Dishes for the isolation of microorganisms. We used a Bold Basal Medium with the addition of sucrose 12g/L for Fungi, Bacto Yeast Extract (BD Company, USA) broth for Heterotrophic bacteria, and solidified Bold Basal Medium for Cyanobacteria and microalgae. Petri dishes were incubated at 21–22°C for heterotrophic bacteria and 25°C for fungi. The growth of colonies was daily checked for heterotrophic bacteria and every 72 hours for Cyanobacteria, microalgae and fungi. Finally, colonies from each dish were axenically picked up and transferred to Petri dishes containing the same culture media used for the isolation of colonies. We used the obtained monoclonal cultures for DNA isolation.

### DNA-based molecular analysis

Genomic DNA was extracted from both biofilm samples and axenic cultivated isolates using the procedure described by [18]. Biofilm samples were amplified for their rRNA genes by PCR using the universal primers for cyanobacteria 16S [19], eukaryote-specific 18S primers for algae [20], 18S and 28S primer for fungi [21].

In the case of cultivated isolates we used the following primer combinations for amplification: ITS1 rDNA for Fungi (ITS1 5′- TCCGTAGGTGAACCTGCGG -3′, [21]; ITS_ADM 5′- TTCAAAGATTCGATGATTCAC -3′); ITS1 [21] and ITS2 rDNA for microalgae [22] and 16S rDNA for Cyanobacteria (16S_long 16Slong_Fw 5’–AGGATGCAAGCGTTATCCG–3’; 16Slong_Rv, 5’–GGGGCATGCTGACTTGACG– 3’).

Similarly, we used different amplification protocols for biofilm samples and cultivated isolates: the formers were carried out on an estimate of 10 ng of extracted DNA, in a final volume of 50 μL containing five μL of 10X PCR buffer, 100 mM of deoxynucleotide triphosphate, 2.5 mM of magnesium chloride, 0.5 mM of primers, and 1U of Taq polymerase (Quiagen, Hilden, Germany). The PCR program consisted of an initial denaturation at 95°C for 4 min and 30 cycles including 1 min of denaturation at 94°C, 45 s of annealing at 56°C, and 2 min extension at 72°C. A final extension of 7 min at 72°C was followed by cooling at 4°C. Cultivated isolates amplifications were carried out in a 25μL aliquot containing approximately 100 ng DNA, a deoxynucleoside triphosphate mixture (0.2 mM each), buffer (1/10 volume of the supplied 10× buffer), supplemented to give a final concentration of 2.5 mM MgCl2, 1.25 U of Taq polymerase (EconoTaq, Lucigen), and 0.5 mM of each primer. The amplification was performed in an Applied Biosystem 2720 thermal cycler. The profile used was 5 min at 95°C, 15 cycles at 95°C for 30s, 55°C for 30s, 72°C for 30s, with annealing temperature increasing by 0.5°C at each cycle, plus 25 cycles with annealing temperature fixed at 55°C and a final elongation step of 7 min at 72°C followed by cooling at 4°C. The sample quality of all PCR products was evaluated through electrophoresis run on 1% (w/v) agarose gel. We obtained Denaturing Gradient Gel Electrophoresis (DGGE) profiles for microbial communities in each sampling site from the PCR products of environmental samples, following the protocol of Nübel et al. [19]. PCR products from cultivated isolates were purified them using QIAquick® PCR Purification kit (Qiagen Inc., Valencia, CA, USA), obtaining sequence reactions with the BigDye Terminator Cycle Sequencing technology (Applied Biosystems, Foster City, CA), and purifying them in automation using the Agencourt CleanSEQ Dye terminator removal Kit (Agencourt Bioscience Corporation, 500 Cummins Center, Suite 2450, Beverly, MA, 01915, USA) and a robotic station Biomek FX (Beckman Coulter, Fullerton, CA). PCR products were analyzed using an Automated Capillary Electrophoresis Sequencer 3730 DNA Analyzer (Applied Biosystems). In both cases, we used the amplification as the sequencing primers. all sequences obtained from molecular analysis were edited using 4Peaks v1.8 software, and nucleotide sequence similarity was determined using BLASTN algorithm v 2.0 (NCBI) (S1 Table).

### Optical, fluorescence and laser confocal microscopy

Aliquots of the biofilm samples collected through the adhesive tape method from each sampling site were observed by optical and fluorescence microscopy (Nikon Eclipse E800) in order to distinguish the structural properties of the microbial communities.

The characterization of three-dimensional biofilm structure was obtained using Leica TCS SP5 (Leica Microsystems CMS GmbH, Mannheim, Germany) Confocal Laser Scanning Microscope (CLSM) equipped with an HCX PL APO 63.0x1.40 oil UV. We took image stacks from each strip of double-sided adhesive tape at 0.50–0.71 μm intervals, and information acquired in the three channels simultaneously (Table 1). The substratum area of the image stack was 1024 x 1024 pixel, with the number of images in each stack varying according to the thickness of the biofilm.

**Table 1:** Fluorescence microscopy information. Red has no labeller because pigments are observed by autofluorescence.

Channel	Wavelength	Emission	Labeller	Marker	Proxy of
Red	633	641–736	-	Pigments (chl *a*, phycobilins)	Phototrophs
Blue	405	415–505	Calcofluor	Bacteria, Hyphae	H. bacteria, Fungi
Green	543	553–636	Concanavalin-A	Extra-Polymeric Matrix (EPS)	EPS itself

We used the open source image processing package Fiji [23]; and also http://www.fiji.sc] for the preliminary preparation of images, according to [24] and the Comstat2 [25] plugin to determine volume and roughness of each Z-stacks [26,27]. Finally, the same plugin was applied to each stack in order to obtain the biomass associated with each color channel (Table 1).

The Z-stacks in which the algal masses showed little and variable autofluorescence were deconvolved and displayed as MIPs (Maximum Intensity Projection). We manually identified and selected cell outlines to measure cell area and mean fluorescence. The Corrected Total Cell Fluorescence (CTCF) was calculated according to [28].

In all the Z-stacks assembled from the swimming pool community images, we detected a marked amount of *Scytonema julianum* filaments emitting weak self-fluorescent signals, probably due to senescence or death. To reduce the effect of dead filaments on measurements the autofluorescence of single *S*. *julianum* cells was equalized using auto-fluorescence values of *Leptolyngbya sp*. cells, this latter being widely present and with a uniform emission of autofluorescence (S1 Fig).

### Data analysis

All the data analysis and visualization were performed using the R environment for statistical computing [29], using the tidyverse collection of packages [30], tidygraph [31] and ggraph [32]. The R markdown file generating tables and figures is available as supplemental material.

The substrates were ordered using the walking order of the rooms, and for each species we added two coarse levels of classification: photo/hetero-trophism and taxa.

We used a Bray-Curtis distance-based nonmetric dimensional scaling (nMDS) to produce an ordination of the communities found at the nine sampling points. Pairwise distance among communities was based on differences among functional groups’ relative richness (i. e. the count of species associated to each group, scaled by the total number of species determined for each site). All the environmental and structural variables measured were projected on the ordination as nonlinear surfaces to indirectly relate them to the ordination axes.

The co-occurrence table that defines the sampled communities was used as a starting point for the assembly of an unweighted, undirected meta-community network that links species based on their contemporary co-occurrence in a site. A layout display was calculated using the Fruchterman-Reingold (FR) force-directed algorithm [33], that defines nodes (*i*.*e*. species) distance as inversely proportional to the number of neighbors a node has *(i*.*e*. the higher species pair co-occurrence frequency is, the lower node distance between them). We then split the meta-community network and sampled communities were plot as single graphs using the calculated FR layout. Finally, we subsetted the bath meta-community based on the three substrates, and displayed every subset as a linear graph, *i*.*e*. nodes were all put on a single line and sorted first by taxon and then by alphabetical order.

## Results

### Environment characterization of sites

The sampling campaign was focused on the three rooms of Suburban Thermae not interested by recent restoration works, where the presence of biofilms was evident at a first glance. The general distribution of biofilms appeared patchy to the naked eye. This is a common feature of microbial lithic communities, depending on variation in substrata characteristics correlated to centimetre scale variations [34].

**Fig 2:**
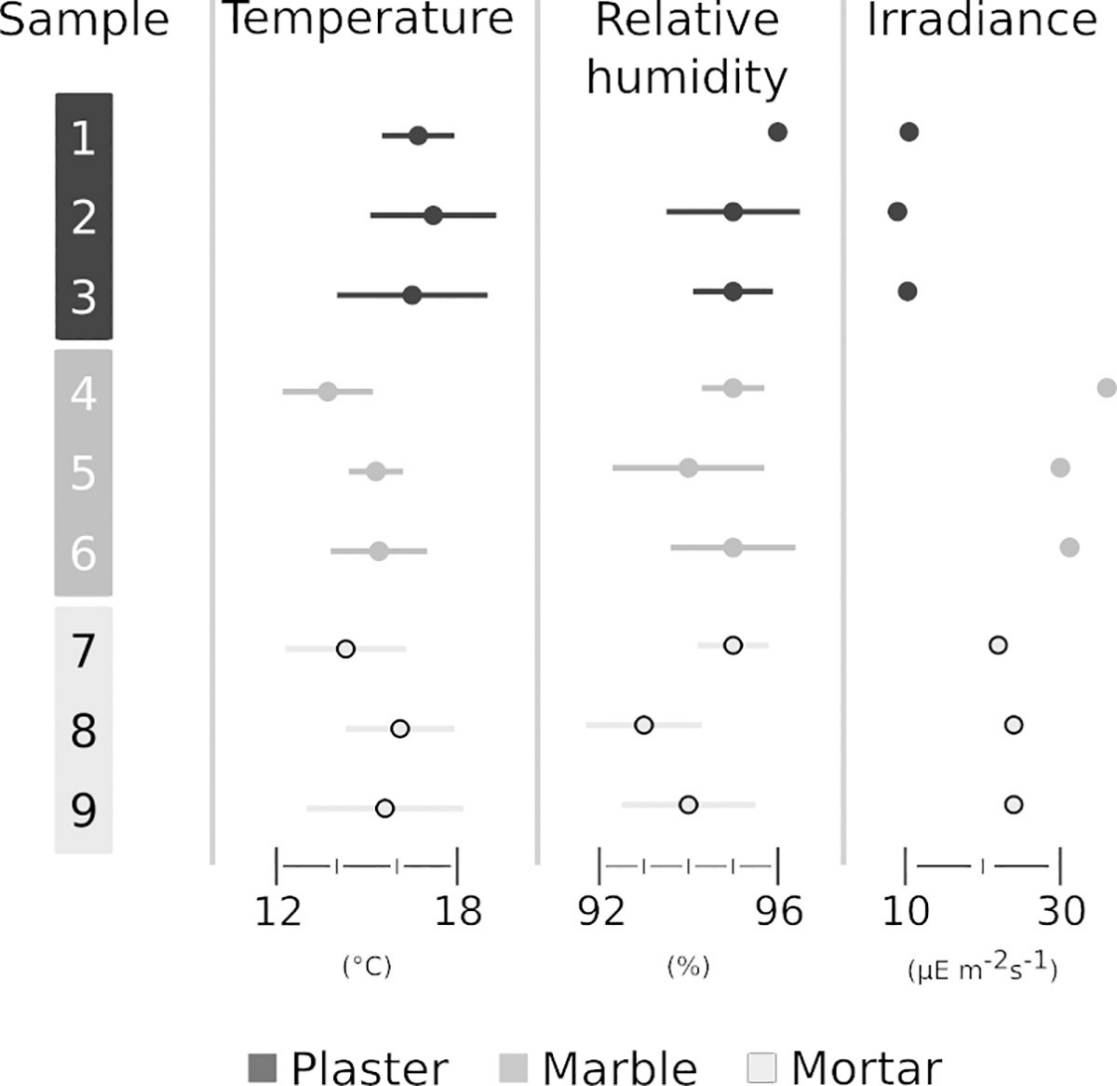
Scatter Plot (SP) of environmental parameters, i.e. of temperature, RH, and irradiance for all the samples of the baths.

Plotting the environmental features of samples (Table 2) shows that relative humidity (RH) is always close to saturation (rh > 92%) and that both humidity and temperature values partially overlaps among samples and substrates, while irradiance changes moving from the plaster substrate towards marble and mortar is lower in the plaster site (Fig 2).

**Table 2:** Mean temperature, relative humidity and light irradiance at the nine sample points. Each value is the mean of three measurements campaigns taken during autumn 2016.

Substratum (room)	Sample (Fig 1A)	Temperature (°C)	pH	Relative humidity (%)	Light irradiance (μE m^-2^s^-1^)
Plaster (*Vestibulum*)	1	16.7±1.2	from 7 to 8	96±0.2	10.5±0.8
	2	17.2±2.1	from 7 to 8	95±1.5	9.0±0.4
	3	16.5±2.5	from 7 to 8	95±0.9	10.3± 1.2
Marble (*Tepidarium*)	4	13.7±1.5	from 7 to 8	95±0.7	36±02
	5	15.3±0.9	from 7 to 8	94±1.7	30±0.6
	6	15.4±1.6	from 7 to 8	95±1.4	31.2±0.9
Mortar (*swimming pool*)	7	14.3±2.0	from 7 to 8	95±0.8	22±0.4
	8	16.1±1.8	from 7 to 8	93±1.3	24±0.7
	9	15.6±2.6	from 7 to 8	94±1.5	24±1.3

### Biofilm richness from molecular data

**Fig 3:**
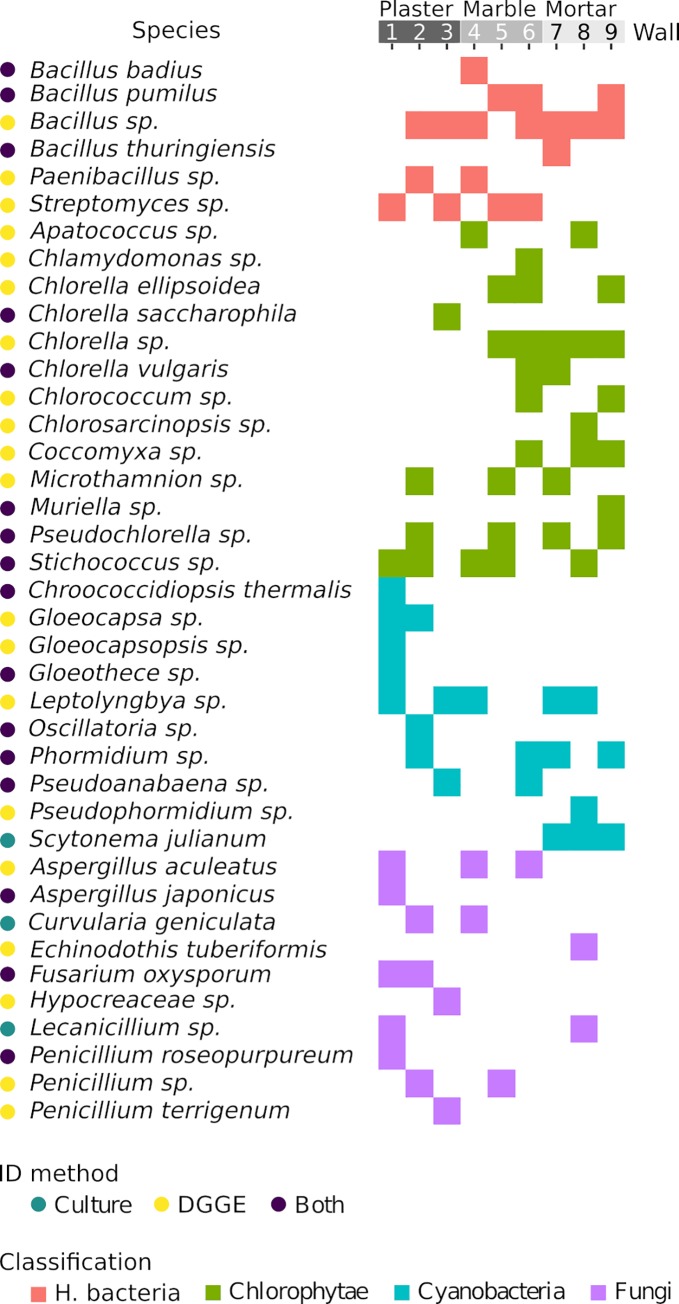
List of the species identified in Herculaneum Suburban Baths samplings. The list is ordered first by classification and then by name. Different square colors stand for different classification, while the dots at the side of species names indicates the ID method used for that species.

The distribution of bacteria, Cyanobacteria, Chlorophyta and Fungi species on each sample from the three selected substrates is shown in Fig 3. Globally, the most represented taxon is Chlorophyta, with thirteen species found, followed by Cyanobacteria and Fungi with ten species, and heterotrophic bacteria, with six species. All the taxa are found at least one time on each substrate, with 50% of Cyanobacteria and Fungi being found only once.

Heterotrophic bacterial profiles were different among substrates, and almost equally distributed. The occurrence of members of *Bacillus* genus was common to all sites, with an unidentified *Bacillus* being the most diffused in the sampled areas. The remaining genera, *Paenibacillus* and *Streptomyces*, were found on plaster and marble but not on the mortar substrate.

The highest number of Cyanobacteria bands was found on plaster, with eight phylotypes, whereas the other two substrates hosted limited Cyanobacterial biodiversity. The only two species common to all three substrates were those of filamentous genera *Leptolyngbya* and *Phormidium*.

We identified thirteen different DGGE bands belonging to Chlorophyta on the three substrates, with dominance from marble and mortar (ten and eleven phylotypes, respectively). The genera most represented belong to *Chlorella* and *Chlorella*-like group. The only genera common to the three substrates were *Stichococcus*, *Pseudochlorella*, and *Microthamnion*.

Finally, we identified DGGE bands belonging to Fungi on the three substrates, with the same dominance pattern of Cyanobacteria. The genera more represented were *Aspergillus* and *Penicillium*, but no species appeared in all substrates.

DGGE failed to spot one cyanobacterium (*Scytonema julianum*) and two microfungi (*Curvularia geniculata* and *Lecanicillium* sp.) in the environmental sample.

Rendering the species list as an alluvial plot make possible the flows of species among substrates, and shows how the species colonizing the marble substrate are also associated to plaster (for Bacteria and Fungi) and mortar (for Cyanobacteria and Chlorophyta). It is worth noting that the Fungi found on the Mortar substrate are unique to that substrate.

### Analysis of CLSM images

After preparation, we analyzed the biofilm samples using a Confocal Light scanning Microscope (S1 Fig). The analysis permitted us to gather insights about biofilms structure and to quantify the relative abundance of and their main autotrophic and heterotrophic components (Table 3).

**Table 3:** Selected CLSM structural parameters of sampled biofilms.

Substrate	Sample	Substratum coverage (%)	Roughness (Ra)	Vol./MIP (μm^3^/μm^2^)	Surface (μm^2^)		Volume (%)
plaster (*Vestibulum*)	1	53.68	2.91	2.85	123912.87	Phototrophs	39.84
						Fungi	1.49
						EPS	58.67
	2	20.88	13.23	8.90	97180.63	Phototrophs	29.49
						Fungi	17.28
						H. bacteria	4.83
						EPS	48.40
	3	10.39	10.48	25.07	54357.94	Phototrophs	73.19
						EPS	26.81
marble (*Tepidarium*)	4	60.07	20.63	9.57	282924.80	Phototrophs	74.77
						Fungi EPS	1.27 23.96
	5	32.58	1.39	0.79	97183.05	Phototrophs	72.49
						Fungi	5.03
						EPS	22.48
	6	37.40	18.58	0.97	217274.49	Phototrophs	98.03
						Fungi	1.97
mortar (*Swimming pool*)	7	50.61	26.16	32.46	559188.97	Phototrophs	52.69
						EPS	47.30
	8	25.15	1.51	2.52	118775.54	Phototrophs	96.21
						Fungi	3.78
	9	6.60	0.93	2.56	27010,71	Phototrophs	77.83
						EPS	22.16

The low intensity of auto-fluorescence with a high Standard Deviation (169.75±96.32) seems to indicate an advanced stage of senescence and limited cellular vitality (S1G–S1I Fig).

Plotting the structural attributes of sampled communities (S2 Fig) shows that the most samples display similar values for surface and volume to MIP ratio; at the same time, roughness and substrate coverage tends to vary among samples. Sample 7 is the one with maximum values for all the attributes.

**Fig 4:**
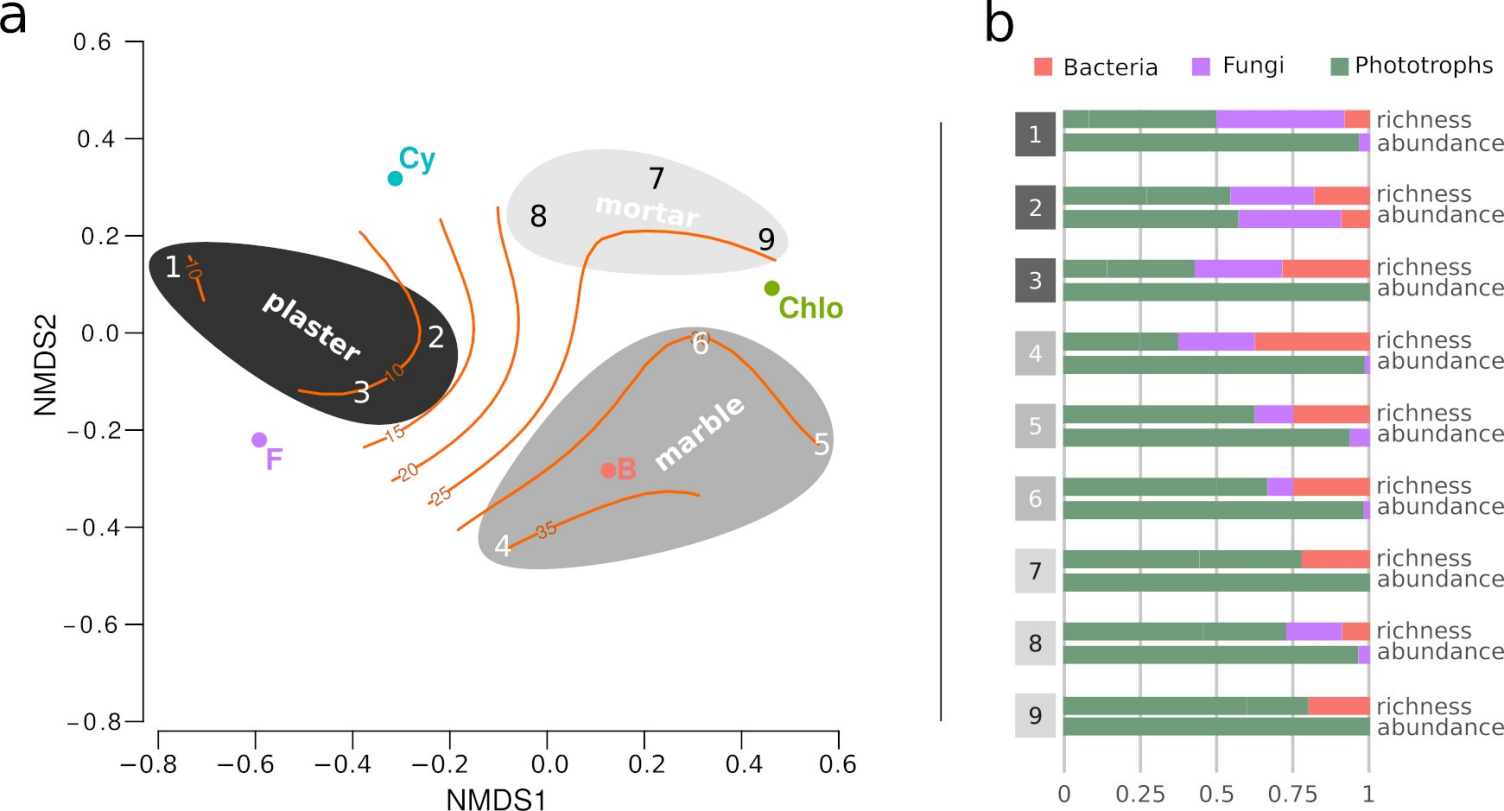
Characterization of microbial community. a) nonmetric dimensional scaling (stress = 0.03) of the communities found at the nine sampling points with respect to the relative taxa count within the different functional groups. Weighted average scores of the four functional groups are displayed as colored dots to ease the interpretation of the ordination, as well as sampling sites and substrates, following the color code presented in Fig 3. The ellipsoids visually cluster sites belonging to the same substrate, while the isolines represent irradiance values (μE m-2s-1); b) Bar plots of relative (%) richness and abundance for all the sampling sites.

The nMDS ordination performed on the nine sites highlight within-substrate similarities (Fig 4A).

Plaster samples are projected at equal distance between Fungi and Cyanobacteria, and display a considerable distance from Chlorophyta and Heterotrophic Bacteria. Mortar communities are characterized by the highest proximity with both Cyanobacteria and Chlorophyta, while marble samples appear to be associated to the maximal Heterotrophic Bacteria contribution to community structure. Projecting the irradiation values over the ordination highlight the light segregation of the plaster communities (all below the 15 μE m^-2^s^-1^ isoline), opposed to the high light condition of the marble ones. Mortar community sits in between. It is worth noting that the Cyanobacteria (and Fungi) to Chlorophyta (and heterotrophic bacteria) gradient is overlapping with the irradiance gradient. The projection of all other variables (both environmental and structural) on the ordination does not highlight any particular trend, and is such reported as supplemental image (S3 Fig).

The calculation of the relative abundance based of the CLSM analysis shows that all the sites are heavily dominated by photoautotroph (>93%, Fig 4B), with the only notable exception of sample 2, where fungi and heterotrophic bacteria represent the 43% of the total. The comparison of this data with the relative richness of the sites based on molecular analyses suggests that fungi and heterotrophic bacteria, while rich in terms of species, are poorly represented in terms of abundance, *i*.*e*. are rare species.

### Co-occurrences based network

**Fig 5:**
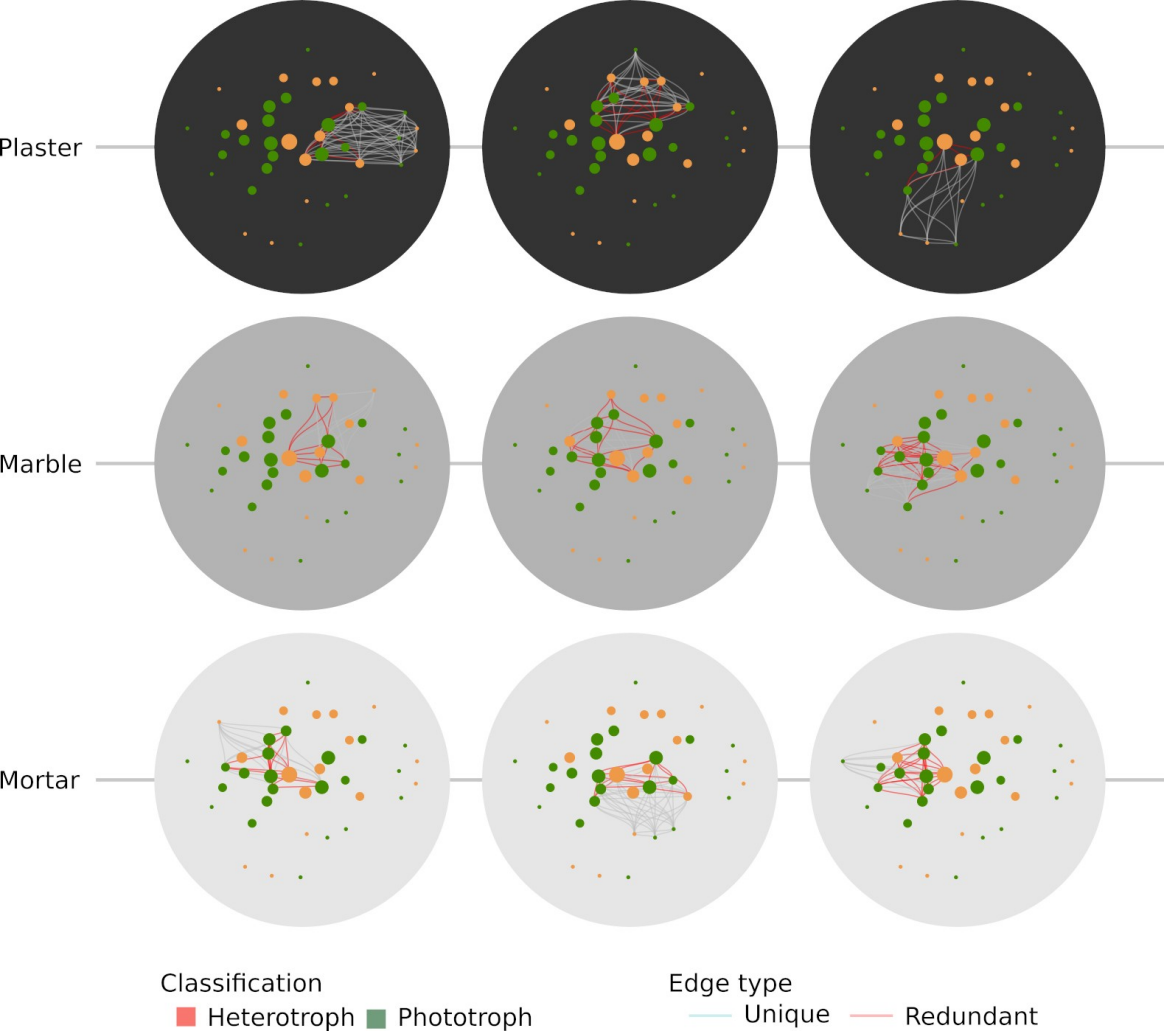
Network display of the communities found in the sampled points of the Herculaneum Suburban Baths using the Fruchterman-Reingold layout (see text). Each circle represent the community found in a single sample. Node diameter is proportional to the number of sites the species is found in, while edge color is red if the species pair is found more than once in any site, and cyan if the species pair is only found in that specific site. The substrates are color-coded as per the other figures.

We created co-occurrence networks for each sampled community and displayed them using the FR layout algorithm (Fig 5). The three plaster communities (1–3, top row in Fig 5) are shifted toward the periphery of the network: such behavior, together with the abundance of local links (cyan edges) indicates that the community is rich in local species. At the other end of the spectrum, the three marble communities (4–6, middle row, Fig 5) tend to be concentrated in the network centre: this, together with the abundance of global links (red edges), indicates that common species represent the backbone of these communities. Finally, the mortar community displays an intermediate behavior, with sample number eight being the richest in local species (cyan connections).

In addition, it should be noted that mortar community displays a high level of promiscuity, *i*.*e*. many species are shared among single communities.

## Discussion

Microorganisms can cause severe damage to historical monuments in different ways, ranging from the corrosion of the building material of the walls to the modifications of surface colors and texture.

Heterotrophic organisms, bio-corrosive chemoorganotrophic bacteria and fungi diversely affect stone and rocks, the rate of corrosion being dependent on the geomorphology of the substratum [9,35]. The relation between lithic facies and species composition of microbial communities has been the focus of different studies, carried out on monuments of different continents. In general, some physical features of the stones, as porosity, roughness and water retention have been associated with the presence of biofilm, and, in some cases, a correlation between microbial species and different types of stone substrates has been reported [5]. Biofilm structure can be dictated also by surface and interface properties o substrata. Bioweathering of limestones and marble used in building construction allows the acquisition of macro and micronutrients from mineral surfaces, along with some organic compounds [35,36]. Plaster and mortars in Roman houses of the Vesuvian cities were basically made of limestones, used as binder, and volcanic materials, as pumices and scoriae [37]. Major and trace elements from Herculaneum mortars evidenced the occurrence of calcium, strontium, sodium and iron [16] that can support microbial life. Also marble shows the same structural components, being composed by limestone crystallized by heat and pressure [38]. The presence of painted walls in the Vestibulum could account for a different mineral availability. Hematite, Egyptian blue, yellow ochre, goethite, and charcoal were the pigments most frequently used to paint walls, and their chemical constituents, together with organic binders occasionally used [39] could represent a supplemental food source for pioneer microbes on the surface of the Vestibulum walls. In this respect, the possible role of micronutrient from substrate in the observed pattern of colonization cannot be ruled out. However, the minor occurrence of heterotrophic bacteria and fungi in all biofilm samples from Herculaneum

While microorganisms which live on the external surfaces of monuments must endure sharp variations of temperature, light intensity and humidity, and are exposed to desiccation and high insolation [40], microbial communities colonizing caves, hypogea, catacombs and similar sites, thrives in quite constant environmental conditions [41,42].

Heterotrophic bacteria and fungi, constitute most of the biodiversity in caves and are ubiquitous in different cave habitats [43,5]; actinobacteria involved in biomineralization processes are common [44,45,46], and *Bacillus* and *Paenibacillus* occurrence has been frequently reported [47,48].

The Suburban Baths of Herculaneum represent an interesting case study of hypogean biofilm communities, in which some critical environmental features such as light, temperature and humidity present limited spatial and seasonal variability and only three different substrates were available for microbial colonization.

The results presented support the description of the Herculaneum baths as an extremely humid and thermally stable environment, where biofilm development can thrive, untouched, due to almost saturating humidity. Our results also indicate that neither species composition nor biofilm structural properties are consistent within the same substrate. This discrepancy could be due to an irradiation gradient that ranges from almost disphotic conditions to dim light, but the possible role of small scale chemical heterogeneity cannot be ruled out due to inevitable limitations of the experimental designed. As a hypogean habitat, the baths host four prominent groups of microorganisms: heterotrophic bacteria, fungi, green algae (Chlorophyta) and blue-green algae (Cyanophyta). All four groups are distributed among the walls in the three colonized sites, with a low contribution of heterotrophic bacterial species to richness, and a considerable, yet heterogeneous, pool of species scattered among substrates.

Actinobacteria belonging to Genus Streptomyces [45,46] and fungi like *Lecanicillium* can occur on different substrates of subterranean environments [47,48]. The Genus Streptomyces has also been found in water, rocks and soils from Spanish and Italian caves [49], and its potential role as a contributor of biotic rock-building processes has been recently proposed [50].

The fungal genera *Aspergillus*, *Penicillium* and *Fusarium*, have a cosmopolitan distribution and are common in many different habitats, including caves, where they respectively represent the first, second and fourth most frequently found taxa [51].

The community composition is seemingly influenced by the irradiation gradient, and goes from being a mix of heterotrophic and dark-adapted autotrophic species (dark composition, the *Vestibulum*) to being rich in low-light adapted autotrophs (dim composition, the other sites), with the *Tepidarium* community sharing its dark-adapted species with the *Vestibulum* and its dim-adapted species with the swimming pool. It is possible to appreciate how the heterotroph/autotroph rates move from the dark vestibule extreme, where fungal richness is maximum, and cyanobacteria steadily compete with green algae, to the dim light swimming pool community, where only *E*. *tuberiformis* and *Lecanicillium* sp. remain as a fungus, and Cyanobacteria are relegated to a small portion of the species pool.

Fungal genera as *Lecanicillium* can occur on different substrates of subterranean environments [52], whereas *Echinodotis tuberiformis* has been described as an insect parasite and an epibiont of different flowering plant species [53,54] and could have reached the Bath with air current, or because of animal/human contamination.

In a recent study carried out on two anthropogenic caves [55], we observed that blue-green unicellular and colonial Cyanobacteria dominate in almost disphotic conditions, whereas filamentous Cyanobacteria showed the highest relative abundance under dim light. In these latter conditions, Chlorophytes and other minor eukaryotic phototrophs also give a relevant contribution to stone surfaces colonization. This trend is also confirmed in Herculaneum Suburban Baths, where unicellular Cyanobacteria genera like *Chroococcidiopsis*, *Gloeocapsa*, *Gloeocapsopsis*, and *Gloeothece* occur prevalently under the very reduced light of the *Vestibulum*, whereas the filamentous *Leptolyngbya* and *Scytonema* are mainly present in the communities from *Tepidarium* and *Swimming pool*.

Our analysis suggests that on the one hand, the meta-communities associated with the three substrates tend to be mostly composed of isolated communities. On the other hand, there is a single meta-community (the *Tepidarium*), sharing almost all of the species with the other two.

The results gathered here support the hypothesis of the Herculaneum baths walls as communities where the low irradiance represents one of the limiting factors, influencing community composition. Indeed, while irradiance gradient influences the three meta-communities, driving the assembly of dark- (*Vestibulum*) and dim light-adapted (*Tepidarium*, *Swimming pool*) communities, the substrate clustering emerging from the nMDS could hint at a contribution of substrate chemistry in driving community assembly.

Using a network depiction permits to appreciate the structure of the communities intuitively; furthermore, building a meta-network gives the chance to explore relationships among the populations. Even with the limit of a co-occurrence matrix, it is possible to gather interesting insights about richness by appropriately choosing the layout algorithm. Our choice of a force-directed algorithm revealed itself fundamental in expressing community structure based on the number of sites where every single species is found. Indeed, the FR-based meta-network layout shows an interesting gradient in community composition, mostly overlapping the walking order of the rooms.

## Conclusion

We have explored the combined effects of substrate and environmental features on microbial community composition in Suburban Baths of *Herculaneum* using molecular and CLSM data to apply numerical ecology tools. Our results indicate that micro-environmental conditions differentiate communities growing on the same substrate, and that it is possible to cluster biofilm morphology using environmental conditions. More specifically, we found that the irradiation gradient is one of the factors that contribute to the differentiation among communities in the Suburban Baths, with the low-light plaster community is rich in fungi and cyanobacteria, while the two dimly lighted communities are richer in Chlorophyceae. In conclusion, our study supports the hypothesis of a strong effect of the environment over substrate in defining community composition, and paves the way to more complex studies, where a higher sampling effort and/or a quantification of microbial abundances could further discriminate community dynamics. Biodeterioration is a prominent issue in conservation of sites and manufacts of archeological interests. Studying biofilm communities through numerical ecological analyses can represent a valuable tool to prevent their proliferation and to develop sustainable approaches to limit or eradicate them.

## Supporting information

S1 Table Accession numbers and percentage of similarity obtained by BLASTn for all the identified taxa, with respect to sampling points, functional group of belonging and method of identification. (TIFF)

S1 Fig Three-channel MIPs of biofilm of the nine sampling points. Plaster (a-c, see also Fig 1E), marble (d-f, see also Fig 1F), mortar (g-i, see also Fig 1G). Scale bar 20 μm.
(TIF)

S2 Fig Scatter Plot of CLSM-determined structural parameters, i.e. roughness, substratum coverage, surface and volume on MIP of the nine sampled biofilms. (TIF)

S3 Fig NMDS and isolines. Environmental (green) and structural (red) measured variables projected on the ordination as nonlinear surfaces for indirect relation to the ordination axes.
(TIF)
