# Supplementary figures and images for: Microbial biofilm community structure and composition on the lithic substrates of Herculaneum Suburban Baths
Source: PLoS One. 2020 May 4;15(5):e0232512. doi: 10.1371/journal.pone.0232512 (PMC7197799; doi:10.1371/journal.pone.0232512)

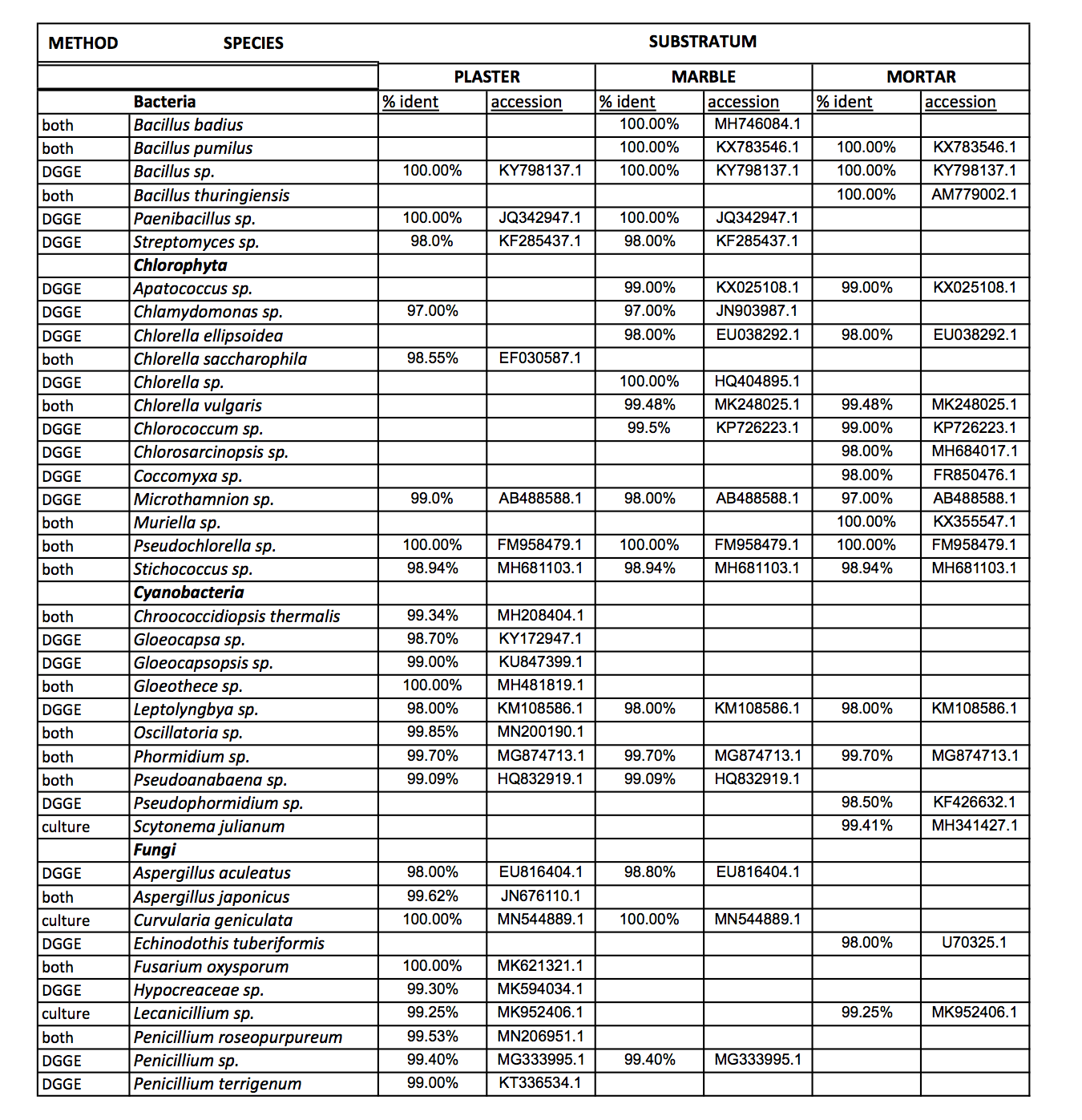

Supplement: S1 Table — (TIFF) [file pone.0232512.s001.tiff]

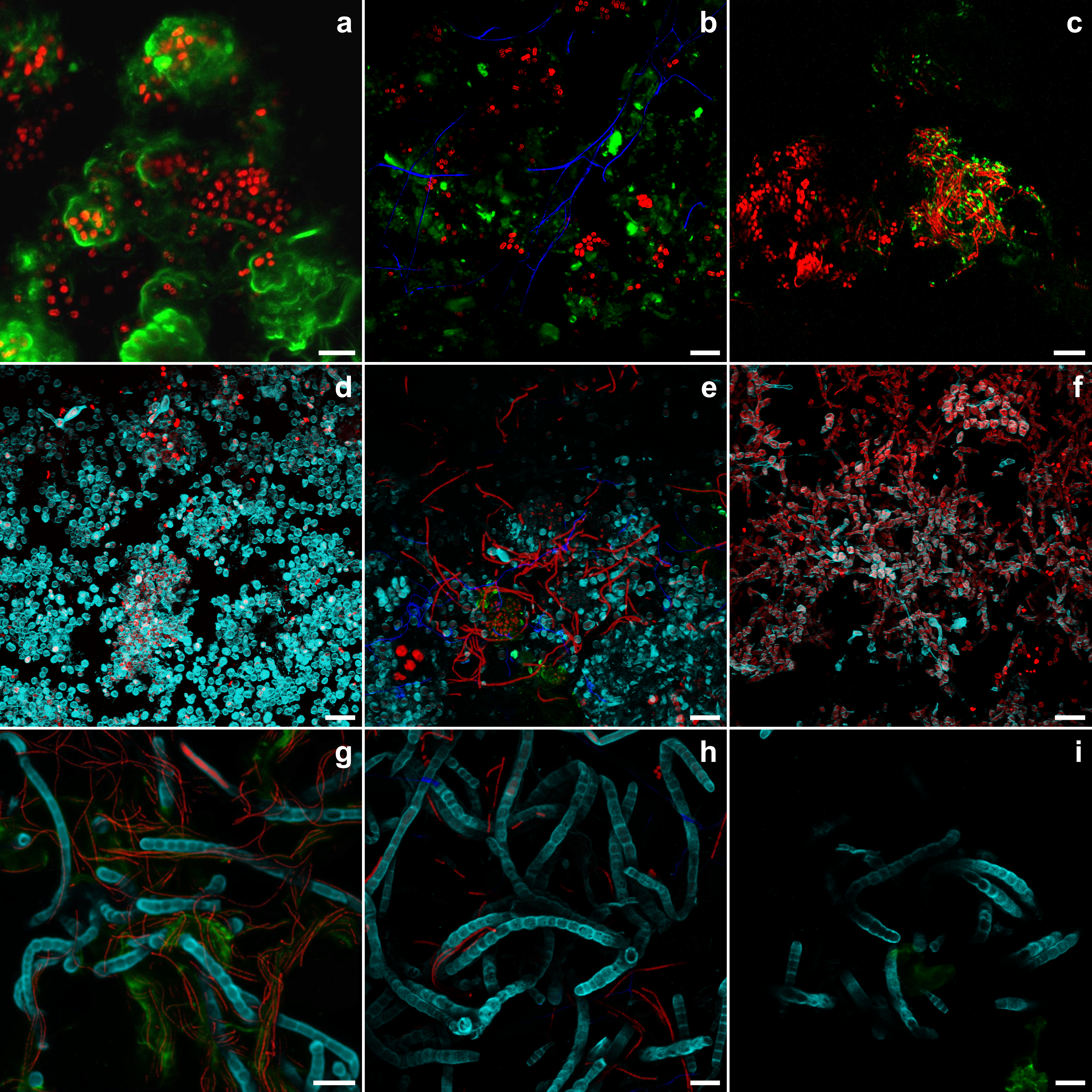

Supplement: S1 Fig — Plaster (a-c, see also Fig 1E), marble (d-f, see also Fig 1F), mortar (g-i, see also Fig 1G). Scale bar 20 μm. (TIF) [file pone.0232512.s002.tif]

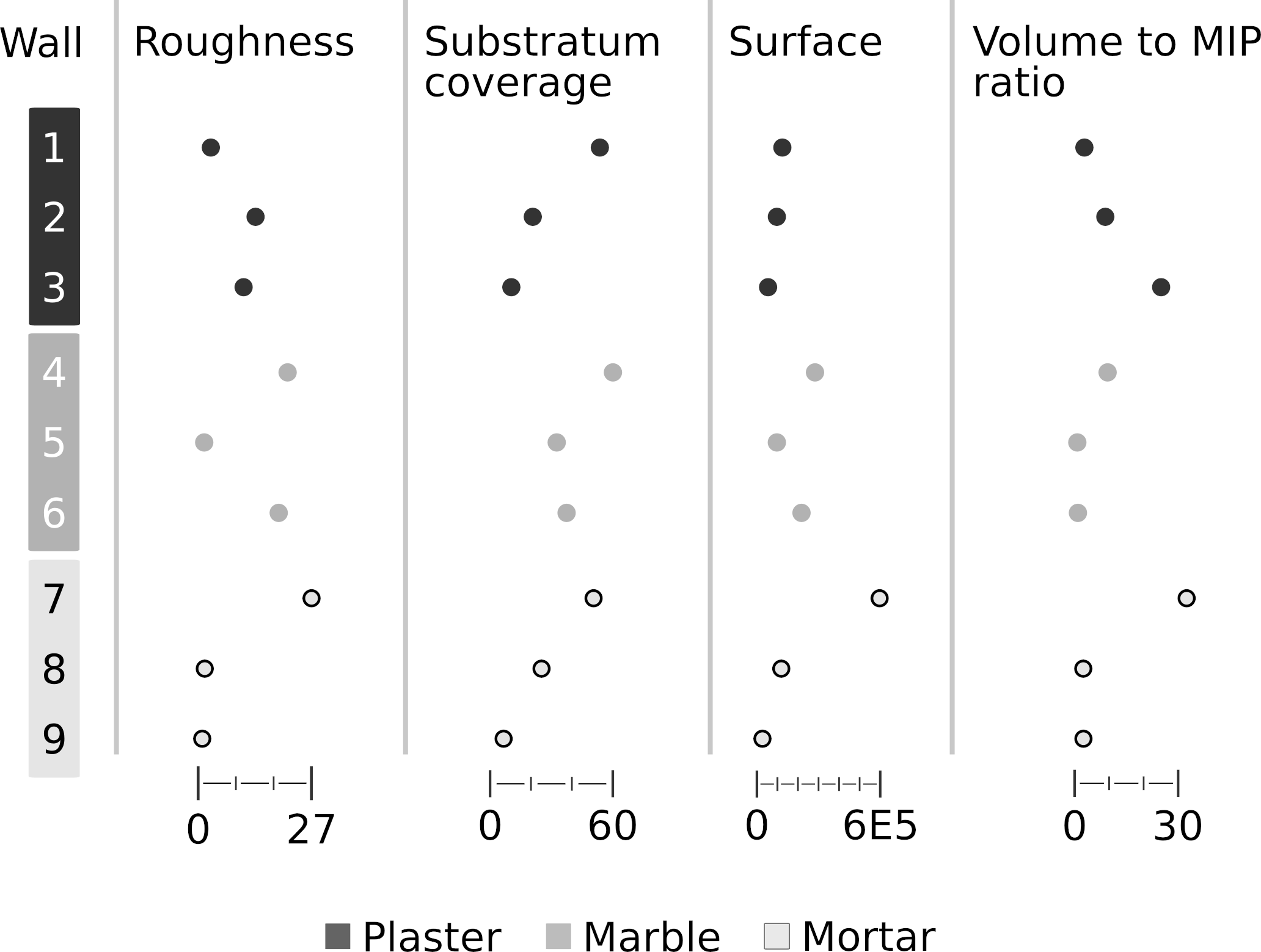

Supplement: S2 Fig — (TIF) [file pone.0232512.s003.tif]

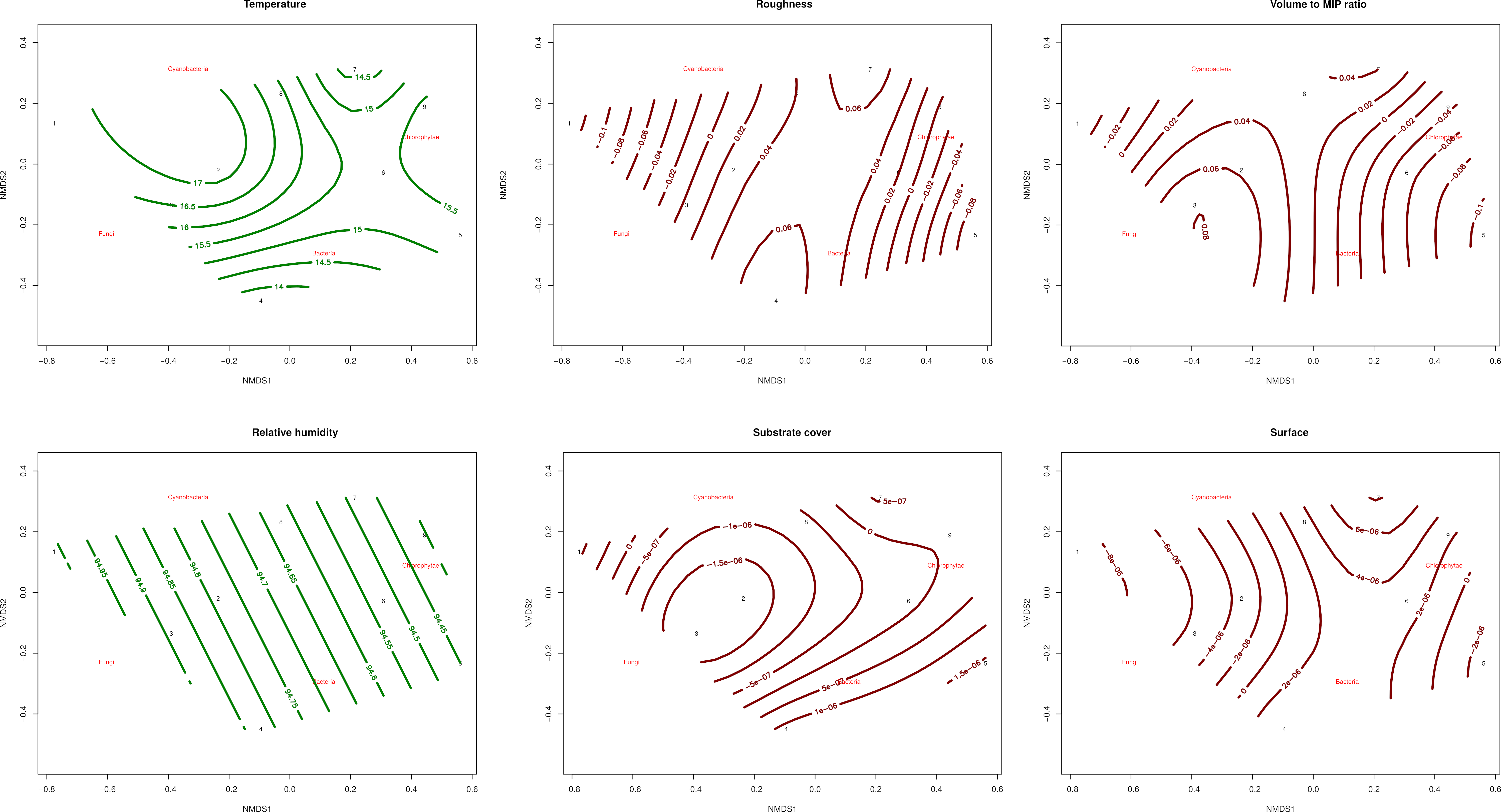

Supplement: S3 Fig — Environmental (green) and structural (red) measured variables projected on the ordination as nonlinear surfaces for indirect relation to the ordination axes. (TIF) [file pone.0232512.s004.tif]
